# S1P receptor 1 signaling reduces arterial thrombosis via up-regulation of endothelial thrombomodulin expression

**DOI:** 10.1126/sciadv.aea9826

**Published:** 2026-07-23

**Authors:** Marcel Benkhoff, Philipp Mourikis, Betül Knoop, Maike Barcik, Tim Huckenbeck, Gabrielle Al-Kassis, Laura Schönfelder, Jasmin Seel, Fernando Kreuz, Moritz Hering, Kajetan Trojovsky, Philipp Wollnitzke, Julia Kielb, Jessica Weber, Thomas Ulrych, Bernhard H. Rauch, Susanne Pfeiler, Lisa Dannenberg, Norbert Gerdes, Tobias Zeus, Malte Kelm, Bodo Levkau, Amin Polzin

**Affiliations:** ^1^ Department of Cardiology, Pulmonology, and Vascular Medicine, University Hospital Düsseldorf, Medical Faculty of the Heinrich Heine University Düsseldorf, Düsseldorf, Germany.; ^2^ Institute of Analytical Chemistry, University of Vienna, Vienna, Austria.; ^3^ Institute for Molecular Medicine III, Heinrich Heine University Düsseldorf, Düsseldorf Germany.; ^4^ Department of Hematology, Oncology, and Clinical Immunology, University Hospital Düsseldorf, Düsseldorf, Germany.; ^5^ Pharmacology and Toxicology, University Medicine Oldenburg, Carl von Ossietzky University Oldenburg, Oldenburg, Germany.; ^6^ CARID, Cardiovascular Research Institute Düsseldorf, Medical Faculty and University Hospital, Düsseldorf, Germany.; ^7^ National Heart and Lung Institute, Imperial College London, London, United Kingdom.

## Abstract

Sphingosine-1-phosphate (S1P) is a key mediator in the cardiovascular system with controversial effects on coagulation. We hypothesized that S1P reduces platelet adhesion and thrombus formation by up-regulating endothelial thrombomodulin (TM), an antithrombotic protein. S1P increased endothelial TM expression via S1P receptor 1 and phosphoinositide 3-kinase signaling. S1P reduced platelet adhesion on endothelial cells in flow-chamber experiments. In the absence of endothelial cells, S1P did not affect platelet activation. In mice, S1P enhanced endothelial TM expression and decreased in vivo arterial thrombus formation but did not change bleeding time. Conversely, sphingosine kinase 1–deficient mice with low S1P concentrations showed reduced endothelial TM expression and enhanced thrombus formation, reversible by TM treatment. In line with this, in an all-comer cohort of 74 patients with cardiovascular disease, higher S1P concentrations were associated with lower circulating thrombin concentrations. In conclusion, S1P inhibited thrombus formation in an endothelium- and TM-dependent manner. This might be a therapeutic target in prevention of thrombus formation without enhancing bleeding risk.

## Teaser

Inhibiting thrombus formation without raising bleeding risk is complex, but endothelial S1P signaling may be a solution.

## INTRODUCTION

Platelet activation and arterial thrombus formation lead to ischemic events such as acute myocardial infarction (AMI) or stroke (*1*). Platelet inhibition is the backbone of treatment and secondary prevention of ischemic events (*2*). However, platelet inhibition is associated with bleeding (*3*). Hence, antiplatelet medication without enhancing bleeding would be desirable. Sphingosine-1-phosphate (S1P) is a bioactive sphingolipid with various functions in cardiovasculature (*4*). However, its effects on platelet reactivity and arterial thrombosis are unclear. Previous studies reported conflicting results (*5*–*7*). The reason for this has not yet been identified. Thrombomodulin (TM) is expressed by endothelial cells (ECs). It can bind and inactivate thrombin. Therefore, TM is a physiological regulator of platelet adhesion (*8*), aggregation, and thrombus formation (*9*). S1P has shown various effects on vascular ECs in terms of permeability and inflammation (*10*). However, the impact of S1P on TM expression and, subsequently, the effect on platelet adhesion and thrombus formation have not yet been investigated. In this study, we hypothesized that S1P reduces platelet adhesion and thrombus formation by up-regulation of endothelial TM. This would identify S1P as an antithrombotic target without increasing bleeding risk.

## RESULTS

**Fig. 1.**
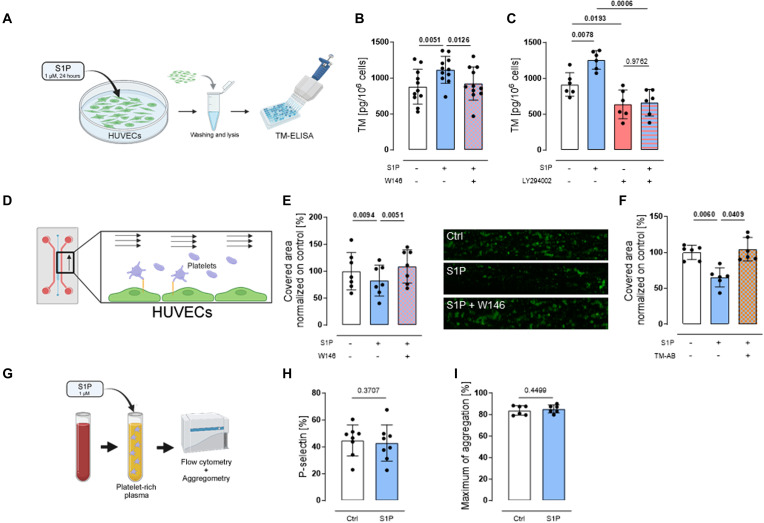
S1PR1 signaling reduces endothelial TM expression. (**A**) HUVECs were treated with S1P for 24 hours. Created in BioRender. Elster, C. (2026) https://BioRender.com/pfgg7hr. (**B**) S1P significantly increased TM expression on HUVECs, which was abolished by S1PR1 inhibition with W146 [*n* = 11, one-way analysis of variance (ANOVA) followed by Tukey’s multiple comparisons test]. (**C**) Inhibition of PI3K by LY294002-blocked S1P-dependent TM expression (*n* = 6, two-way ANOVA followed by Tukey’s multiple comparisons test). (**D**) Platelet adhesion was analyzed in flow chamber experiment with treated HUVECs. Created in BioRender. Elster, C. (2026) https://BioRender.com/pfgg7hr. (**E**) S1P treatment decreased platelet adhesion to ECs. W146 nullified this effect by S1PR1 inhibition (*n* = 7, one-way ANOVA followed by Tukey’s multiple comparisons test). (**F**) To further investigate TM dependency of these effects, we evaluated platelet adhesion to ECs in the presence of TM neutralizing antibody. TM antibody (TM-AB) reversed S1P antiadhesive effects (*n* = 6, one-way ANOVA followed by Tukey’s multiple comparisons test). (**G**) To analyze the impact of S1P on platelets in the absence of ECs, PRP was used. Created in BioRender. Elster, C. (2026) https://BioRender.com/pfgg7hr. (**H**) P-selectin expression on platelets upon thrombin receptor activating peptide-6 (TRAP-6) activation was unchanged after incubation with S1P (*n* = 8, paired *t* test). (**I**) Platelet aggregation upon TRAP-6 activation was unaltered by S1P (*n* = 6, paired *t* test). Ctrl, control.

To analyze the impact of S1P on ECs, we incubated human umbilical vein endothelial cells (HUVECs) with S1P (Fig. 1A). We found that S1P significantly increased TM expression on HUVECs by 25%. This effect was abolished by S1P receptor 1 (S1PR1) inhibition with W146 (Fig. 1B). However, at this time point, no changes in mRNA expression were observed (fig. S1A). In addition, similar changes in soluble TM could be observed in the HUVEC supernatant, suggesting no changes in TM shedding (fig. S1, B and C). Further analyses on the downstream signaling revealed that phosphoinositide 3-kinase (PI3K) inhibition blunted S1P-dependent TM expression (Fig. 1C). Next, we investigated the effect of S1P on platelet adhesion (Fig. 1D). S1P decreased platelet adhesion to ECs. This applies to both HUVECs displaying an endothelial phenotype (Fig. 1, F and G) (*11*) and human aortic endothelial cells (HAECs) (fig. S2). This effect was abolished by S1PR1 inhibition (Fig. 1E) and neutralizing TM antibody (Fig. 1F). However, S1P did not affect platelet activation in the absence of ECs (Fig. 1G). Neither platelet-p-selectin expression nor platelet aggregation upon activation was altered after S1P incubation (Fig. 1, H and I, and fig. S3).

**Fig. 2.**
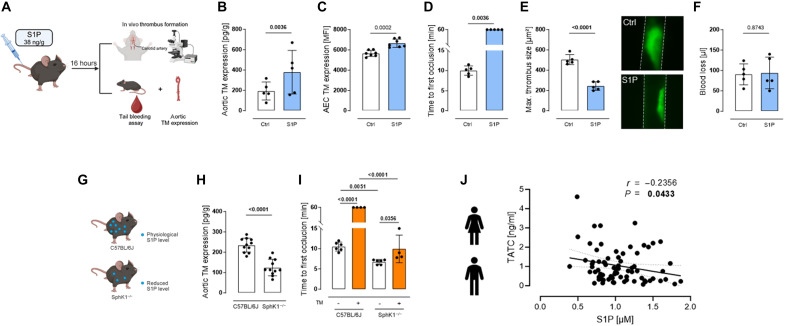
Enhanced S1P-dependent TM expression reduced in vivo thrombus formation without increased bleeding risk. (**A**) Overview of murine experiments. Created in BioRender. Elster, C. (2026) https://BioRender.com/pfgg7hr. (**B**) TM expression of the aorta was increased in C57BL/6J mice that were treated with S1P (38 ng/g) 16 hours before (*n* = 5, unpaired *t* test). (**C**) Moreover, TM expression of aortic ECs (AECs), determined by flow cytometry, was increased in C57BL/6J mice that were treated with S1P (38 ng/g) 16 hours before (MFI, mean fluorescence intensity; unpaired *t* test, *n* = 7). (**D**) S1P inhibited in vivo carotid arterial thrombus formation in mice. Occlusion of the vessel could not be detected during the 1-hour recording. (**E**) Maximal thrombus size was reduced in S1P-treated animals (*n* = 5, unpaired *t* test). Exemplary images are provided. (**F**) In these mice, bleeding was not enhanced (*n* = 5, unpaired *t* test). (**G**) To analyze low S1P level, SphK1-deficient mice were analyzed (SphK1^−/−^). Created in BioRender. Elster, C. (2026) https://BioRender.com/pfgg7hr. (**H**) In SphK1-deficient mice, TM expression was significantly lower in comparison to wild-type mice (*n*_C57BL/6J_ = 11, *n*_SphK1−/−_ = 11, unpaired *t* test). (**I**) TM inhibited in vivo carotid arterial thrombus formation in mice. Occlusion of the vessel could not be detected during the 1-hour recording. In contrast, SphK1^−/−^ mice with low S1P levels showed increased thrombus formation. Treatment with TM in these animals was able to reverse the enhanced thrombus formation (*n*_C57BL/6J_ = 6, *n*_C57BL/6J+TM_ = 4, *n*_SphK1−/−_ = 6, *n*_SphK1−/−+TM_ = 4, two-way ANOVA followed by Tukey’s multiple comparisons test). (**J**) In 74 patients with cardiovascular disease, plasma S1P was negatively associated with plasma thrombin concentrations [measured by thrombin-antithrombin complex (TATC), Pearson’s *r* = −0.2356, *P* = 0.0433].

To assess the in vivo effects of our findings, we first investigated TM expression in mice that were treated with S1P (Fig. 2A). We found increased TM expression in whole aortic tissue after S1P treatment (Fig. 2B). Flow cytometry analyses showed a specific increase in TM expression in the aortic endothelium (Fig. 2C). This was confirmed in histological analyses (fig. S4). The increase of aortic TM expression led to reduced in vivo thrombus formation as no occlusion and reduced maximal thrombus size could be observed in S1P-treated animals (Fig. 2, D and E). S1P treatment did not enhance bleeding in these animals (Fig. 2F). To reflect the impact of low S1P levels, we additionally analyzed sphingosine kinase 1 (SphK1)–deficient mice, which have about 70% less circulating S1P in comparison to wild-type mice (Fig. 2G) (*12*). In these mice, we found reduced TM expression in the aorta (Fig. 2H). This was associated with enhanced thrombus formation (Fig. 2I). Treatment with TM in these animals was able to reverse this effect (Fig. 2I). To examine our previous data translationally, we included 74 patients with cardiovascular diseases (table S1). They were 72.5 ± 13.4 years old, one third were male, and they had a body mass index of 26.55 ± 5.47. Medication use was consistent with a cardiovascular high-risk cohort. Fifty-five percent were on statin therapy and 51% on oral anticoagulation (detailed patient characteristics are presented in table S1). S1P plasma concentrations and circulating thrombin (measured by thrombin-antithrombin complexes) were determined. In line with this, we found an association between S1P and circulating thrombin. High S1P levels were associated with low thrombin concentrations (Fig. 2J).

## DISCUSSION

**Fig. 3.**
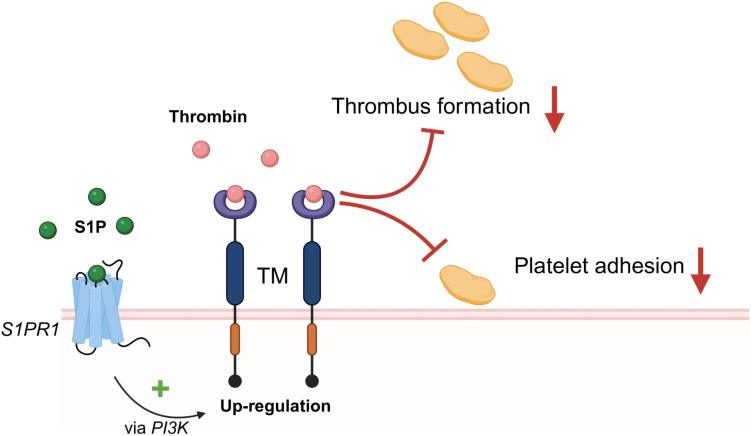
Main findings of this study. Created in BioRender. Elster, C. (2026) https://BioRender.com/io2y714. S1P inhibits platelet adhesion and thrombus formation in an endothelium- and TM-dependent manner. Mechanistically, activation of S1PR1 and PI3K mediates TM up-regulation.

The major findings of this study were as follows: (i) S1P enhances endothelial TM via S1PR1 and PI3K, and (ii) this leads to reduced platelet adhesion and arterial thrombus formation (iii) without enhanced bleeding (Fig. 3). We present here a previously unknown mechanistic link between sphingolipids and ECs. S1P treatment led to a significant increase in protein expression, but analysis of mRNA expression using quantitative polymerase chain reaction did not reveal any corresponding changes. This suggests that the regulation of the protein under investigation occurs predominantly at the posttranscriptional level. Alternatively, a temporary induction of mRNA expression is conceivable, which was no longer detectable at the time of analysis, while protein expression remained elevated.

It is known that S1P contributes to endothelial integrity (*13*) and glycocalyx (*14*). We now found an antithrombotic effect of S1P by up-regulation of endothelial TM expression. This has several implications.

First, it might explain previous inconsistent findings of studies investigating S1P effects on platelets. Early reports demonstrated that very high, but supraphysiological concentrations of S1P (>10 μM) can induce platelet activation (*6*). In contrast, S1P in physiological dosages was unable to induce platelet aggregation in platelet-rich plasma (PRP) (*7*). However, S1P was shown to reduce thrombus formation in vivo (*15*). Previously, attempts were made to explain this unclear data situation with different S1P dosages, species studied, choice of platelet agonists, and static versus flow-based assay conditions (*5*). In our study, only in the presence of ECs, S1P had antithrombotic properties. Moreover, our data are in line with additional previous studies that revealed (i) increased platelet adhesion and arterial thrombosis in SphK1-deficient mice (*16*), (ii) inhibition of arterial thrombosis in SphK2-deficient mice with enhanced S1P levels (*15*), and (iii) no effect of S1P on platelet activation ex vivo (*17*, *18*). These conflicting results can now be explained by our findings of S1P regulating endothelial TM.

Second, it reveals S1P as potential therapeutic target in AMI. We and others have previously described cardioprotection by S1P in acute coronary syndrome. S1P improves infarct size and cardiac function after AMI (*19*, *20*). Beyond that, lower S1P concentrations are associated with increased mortality in patients with AMI (*12*). The presented findings underline a promising role of S1P in AMI. It not only addresses improvement of cardiac function and reduction of infarct size, but also exerts antithrombotic effects. However, our findings are based on experimental in vitro and murine in vivo models, complemented by an associative analysis in a patient cohort. Therefore, we did not establish clinical efficacy or safety but strengthened the promising role of S1P in AMI.

Third, currently, primary prevention of ischemic events with antiplatelet agents is not recommended (*21*). This is due to the risk of bleeding by antithrombotic medication. Novel factor XIa (FXIa) inhibition did not prove efficient in reduction of ischemic events in atrial fibrillation patients (*22*). A possible explanation might be missing antiplatelet effects by FXIa inhibition as compared to FXa inhibitors (*23*). Here, we present S1P as antithrombotic agent without enhanced bleeding risk. Hence, S1P might be a previously unidentified therapeutic target in primary prevention of ischemic events in high-risk patients as well. Further validation in translational and clinical studies is required. Global S1P signaling is known for its pleiotropic effects, including immune modulation, effects on lymphocyte trafficking, and possible alterations in vascular permeability and endothelial barrier function (*24*, *25*). For this reason, careful consideration must be given in the future to which S1P modulators can be used for the prevention of ischemic events. Specific and, ideally, endothelium-specific S1PR1 modulators are certainly preferable to classic and nonselective S1P modulators such as fingolimod.

This study has some limitations. First, we have to acknowledge that the carotid artery injury model, although well established, is known to exhibit a certain degree of variability and may not fully reflect all mechanisms of thrombosis. In particular, results obtained from this model may not be directly comparable to those derived from alternative thrombosis models, such as laser-induced arteriolar injury. Therefore, our findings should be interpreted within the context of this specific experimental system. Second, given the lack of an a priori power calculation, we performed post hoc power analyses for the key in vivo endpoints. For whole aortic TM expression, the calculated statistical power is below the commonly accepted threshold of 0.8, indicating that this analysis may be underpowered. However, flow cytometry analyses of endothelial TM expression showed a statistical power >0.9. In addition, the in vivo thrombus formation experiments, used as functional endpoints, demonstrated high statistical power (>0.99) for both thrombus size and time to first occlusion, supporting the robustness of these findings. Third, global depletion of S1P in SphK1-deficient mice may, in principle, affect multiple cell types, including immune cell subsets, which could indirectly influence thrombus formation. However, prior studies have extensively characterized immune cell composition and inflammatory profiles in SphK1-deficient mice and reported no major baseline immune activation or skewing that would explain the observed prothrombotic phenotype (*15*, *16*, *26*, *27*). Moreover, the evidence of our additional ex vivo and in vitro data support an endothelial TM-S1P axis as the dominant mechanism underlying the observed phenotype. In conclusion, S1P/S1PR1-mediated up-regulation of endothelial TM represents a mechanistic insight and a potential therapeutic concept to prevent arterial thrombosis without the risk of bleeding.

## MATERIALS AND METHODS

### Cell culture

HUVECs (Promocell, no. C-12203) were seeded into a flask (Cellstar) filled with growth medium (2% fetal calf serum, Promocell) including 1% penicillin/streptomycin (Thermo Fisher Scientific). Cells were split every 3 days to fibronectin-coated 12-well plates (Cellstar). Fibronectin-coated slides were used because fibronectin is a key component of the subendothelial extracellular matrix and supports stable endothelial adhesion, spreading, and physiologically relevant integrin signaling. Compared with collagen or gelatin, fibronectin promotes homogeneous endothelial monolayer formation and mechanical stability under flow, minimizing nonspecific platelet-matrix interactions. Experiments were conducted with a cell density of 80%. Incubation with S1P (1 μM, Sigma-Aldrich) was performed for 24 hours. If required, S1PR1 antagonist W146 (10 μM, Tocris) or PI3K inhibitor LY294002 (20 μM, Sigma-Aldrich) were added 15 min prior to S1P application. After 24 hours, TM expression of HUVECS and the corresponding supernatant was measured by human TM enzyme-linked immunosorbent assay (ELISA) Kit (Abcam) according to the manufacturer’s protocol.

### Platelet adhesion

HUVECs were seeded onto fibronectin-coated flow chambers (Sigma-Aldrich). Laminar flow of 2 dyne/cm^2^ for 72 hours was used to ensure homogeneous distribution of cells and to display endothelial phenotype (*11*). After washing, S1P (1 μM), W146 (10 μM), and TM antibody (dilution 1:1000; ab109189, Abcam) were added. Citrate-anticoagulated whole blood of healthy volunteers was stained with calcein (4 μM, Sigma-Aldrich) and perfused through the capillary for 10 min at 10 dyne/cm^2^. In addition, HAECs (Promocell, no. C-12271) were used. Laminar flow of 10 dyne/cm^2^ for 24 hours was applied before whole blood was perfused as described above. Pictures were taken using the Nikon Eclipse TE2000-U microscope, and results were calculated using Bioflux-Montage software. Results were given in platelet-covered area in percent normalized on untreated controls.

### Platelet activation assays

PRP was generated from citrate-anticoagulated whole blood after centrifugation at 270*g* for 10 min. PRP was incubated with or without S1P (1 μM) for 6 min. Either thrombin receptor activating peptide-6 (10 μM, Sigma-Aldrich), adenosine diphosphate (2.5 μM, Sigma-Aldrich), or collagen-related peptide (0.05 μg/ml, Sigma-Aldrich) were used to induce platelet activation. For p-selectin expression, anti-CD41 antibody (Emfret Analytics, M023-2) and anti-CD62 antibody (Santa Cruz Biotechnology, sc-8419) were used. Flow cytometry was performed with Guava easyCyte 8HT Benchtop Flow Cytometer. Results were calculated using FlowJo software. For aggregation, PRP was used in aggregometry (APACT 4004 LABiTec) as previously described (*12*).

### Animals

C57BL/6J wild-type mice were received from Janvier Labs (Saint-Berthevin, France). SphK1-deficient mice (SphK1^−/−^) were bred as previously described (*28*). Male mice at the age of 12 to 15 weeks were used for this study. All animals had a free access to standard chow and drinking water ad libitum. The mice were kept on a 12-hour day-night rhythm. The animals were not operated and analyzed in blocks, but in a random order to minimize possible confounders. Investigators were blinded. An a priori sample size calculation could not be performed due to the pilot nature of the study. If required, mice were treated with S1P via tail vein injection (38 ng/g) (*29*). Mice were randomly divided into control and treatment groups. No mice were excluded from the analyses. We used the ARRIVE checklist when writing our report (*30*).

### Murine whole aorta TM determination

Mice were sacrificed at the age of 12 weeks. Aortic TM expression was determined via Mouse THBD/CD141 TM ELISA Kit (Life Span Bioscience) according to the manufacturer’s protocol. Aortic TM expression was calculated as mass of TM per mass of aorta.

### Detection of TM expression on aortic ECs

Explanted aortae were cut in 2-mm pieces, digested using an enzyme mix [collagenase I (400 U/ml), collagenase XI (120 U/ml), hyaluronidase I-S (60 U/ml), deoxyribonuclease I (60 U/ml), and 20 mM Hepes diluted in Dulbecco’s Balanced Salt Solution (DPBS) with Ca^2+^ and Mg^2+^] for 60 min at 37°C and 600 rpm shaking, and then filtered through a 100-μm cell strainer to generate a single-cell suspension. Aortic cells were washed with DPBS and stained for flow cytometry analysis, using fluorophore linked anti-CD45 (BD, clone: 30-F11), CD31 (BioLegend, clone: 390), and CD141 (Miltenyi Biotec, clone: REA964) antibodies and ViaKrome808 as viability dye, for 30 min at 4°C. Flow cytometry was performed with a Beckman Coulter CytoFLEX LX Flow Cytometer, and results were calculated using FlowJo software.

### In vivo arterial thrombus formation

After 5 days of daily treatment with TM (0.4 g/kg intraperitoneally), arterial thrombosis was induced by vessel injury of the carotid artery by using an Fe(III) chloride–soaked tissue as described previously (*31*). For this experiment, recombinant soluble TM (Thermo Fisher Scientific, no. 100-58) was used. It contained the extracellular domain of TM, and its effect is mediated via functional thrombin neutralization and protein C activation, rather than reconstitution of endothelial TM signaling.

### Tail bleeding assay

Mice were anesthetized with ketamine (100 mg/kg and xylazine (10 mg/kg) and placed on a 37°C heating plate in a prone position. After amputation of a 5-mm segment of the tail tip, the tail was dipped in a tube filled with 37°C prewarmed isotonic saline. Blood was collected for half an hour. To analyze the bleeding volume, hemoglobin concentration was measured spectrophotometrically at 550 nm as previously described (*31*). Blood loss was calculated from a standard curve, which was obtained by lysing defined volumes of mouse blood.

### Patients

Information on the patients examined, including their characteristics and the blood analyses performed, can be found in the Supplementary Materials.

### Statistical analysis

Statistical analyses were conducted using GraphPad Prism statistical software (GraphPad Software Inc.). Normally distributed continuous variables were analyzed using *t* test and nonnormally distributed variables using Mann-Whitney *U* test. Comparison of three or more groups was performed using analysis of variance (ANOVA) or mixed-effects analysis as appropriate. Comparisons between single groups were performed using Tukey’s multiple comparison test. *P* values below 0.05 were defined significant.

### Ethics committee approval

All mice experiments were approved by Landesamt für Natur, Umwelt und Verbraucherschutz Nordrhein-Westfalen (LANUV; 81-02.04.2020.A408) and in accordance with the European Convention for the Protection of Vertebrate Animals used for Experimental and Other Scientific Purposes (Council of Europe Treaty Series no. 123) and 2010/63/EU. The human study was conducted with the approval of the Institutional Ethics Committee (Medical Faculty, Heinrich Heine University, Düsseldorf, ID: 2019035018) and in accordance with the World Medical Association Declaration of Helsinki.

## Supplementary Materials

### This PDF file includes:

Supplementary Methods Table S1 Figs. S1 to S4 References
