# Supplementary material for: S1P receptor 1 signaling reduces arterial thrombosis via up-regulation of endothelial thrombomodulin expression
Source: Sci Adv. 2026 Jul 23;12(30):eaea9826. doi: 10.1126/sciadv.aea9826 (PMC13394366; doi:10.1126/sciadv.aea9826)
Supplement: Supplementary file 1 — Supplementary Methods Table S1 Figs. S1 to S4 References [file sciadv.aea9826_sm.pdf]

Science Advances
AAAS

Supplementary Materials for

S1P receptor 1 signaling reduces arterial thrombosis via up-regulation of endothelial thrombomodulin expression

Marcel Benkhoff *et al.*

Corresponding author: Amin Polzin, amin.polzin@med.uni-duesseldorf.de

Sci. Adv. **12**, eaea9826 (2026)
DOI: 10.1126/sciadv.aea9826

This PDF file includes:

Supplementary Methods
Table S1
Figs. S1 to S4
References

Methods (extended)

Patients

We conducted a hypothesis generating, prospective, monocentric, translational analysis in 74 patients with cardiovascular disease. An all-comers design was applied. Inclusion criteria were age ≥ 18 years, occurrence of one cardiovascular disease and written informed consent. Exclusion criteria were malignant comorbidities and coagulopathies. Written informed consent was obtained from all participants. The study was conducted with an approval of the Institutional Ethics Committee (Medical Faculty, Heinrich Heine University, Düsseldorf, ID: 2019035018) and in accordance with the World Medical Association Declaration of Helsinki. Blood sampling was conducted as recommended by the manufacture of Human Thrombin-Antithrombin Complex (TATC) ELISA Kit (Abcam) as TATC was determined via this ELISA Kit. Plasma S1P content was determined by LC-MS/MS analysis.

LC-MS/MS analysis

Analysis of sphingolipids was performed as previously described by Peng et al. (*32*) with minor modifications. Briefly, a Vanquish Flex UHPLC system (Thermo Fisher Scientific) was equipped with an Ascentis Express C18 main column (150 mm × 2.1 mm, 2.7 µm, Supelco) and fitted with a guard cartridge (50 mm × 2.1 mm, 2.7 µm, Supelco) in a column oven with a temperature of 60 °C. Solvent A was ACN/H_2O (6:4, v:v; 10 mM AF, 0.1 % FA, 5 µM PA), and solvent B was IPA/ACN (9:1; v:v; 10 mM AF, 0.1 % FA, 5 µM PA). The separation was carried out at a flow rate of 0.5 ml/min with the following 25 min long gradient: initial (30 % B), 0.0–2.0 min (hold 30 % B), 2.0–3.0 min (30–56.1 % B), 3.0–4.0 min (56.1–58.3 % B), 4.0–5.5 min (58.3–60.2 % B), 5.5–7.0 min (60.2–60.6 % B), 7.0–8.5 min (60.6–62.3 % B), 8.5–10.0 min (62.3–64.0 % B), 10.0–11.5 min (64.0–64.5 % B), 11.5–13.0 min (64.5–66.2 % B), 13.0–14.5 min (66.2–66.9 % B), 14.5–15.0 min (66.9–100.0 % B), 15.0–19.0 min (hold 100 % B), 19.0 min (5 % B), 19.0–22.0 min (hold 5 % B), 22.0 min (30 % B), 22.0–25.0 min (hold 30 % B). The LC system was coupled to a QTRAP 6500+ (Applied Biosystems). The measurements were performed in positive mode with the following ESI source settings: curtain gas 30 arbitrary units, temperature 250 °C, ion source gas I, 40 arbitrary units; ion source gas II 65 arbitrary units, collision gas medium; ion spray voltage +5500 V, declustering potential +100 V, entrance potential +10 V, and exit potential +13 V. For the scheduled SRM, Q1 and Q3 were set to unit resolution. The scheduled SRM detection window was set to two min, and the cycle time was set to 0.5 s. Data was acquired with Analyst (version 1.7.2; AB Sciex) and Skyline (*33*) was used to visualize results and manually integrate signals.

Real time polymerase chain reaction (PCR)

HUVECs were examined by PCR. RNA was isolated with RNeasy Mini Kit (Qiagen). After that, isolated RNA was reverse transcribed with SuperScript IV VILO Master Mix with ezDNase synthesis kit (Thermo Fisher). Next, PCR analysis was performed with TaqMan Fast Advanced Master Mix and manufactured primers GAPDH (Hs03929097_g1) and THBD (Hs00264920_s1; all Thermo Fisher). Finally, data were analyzed and presented as x-fold change via $2^{-\Delta\Delta CT}$ method.

Histological assessment of TM

Mice were sacrificed 16 h after treatment and perfused with cold PBS. Aortae were then removed and fixed in 4% paraformaldehyde (PFA, Alfa Aesar, Massachusetts, USA) overnight. Hearts were then dehydrated and embedded in Tissue-Tek O.C.T. Compound (Tissue-Tek, Sakura Finetek, Umkirch, Germany). Aortae were cut in 5 µm sections using a cryotome (Leica CM3050 S). After rehydrating with PBS, tissue was treated with blocking buffer (2.5% goat serum in PBS) for 60 min to prevent non-specific binding, followed by incubation with primary antibody (Rb anti-thrombomodulin (ab230010, abcam, Cambridge, UK)) overnight at 4°C. After washing with PBS, secondary antibody (AlexaFluor 488 goat anti-rabbit IgG (A-32731, Invitrogen, Darmstadt, Germany)) was incubated for 1h at RT and second primary antibody (Rat Anti-CD31 (ab256569, abcam, Cambridge, UK)) overnight at 4°C. The next day secondary antibody (AlexaFluor 555 goat anti-rat IgG (H+L)(A-21434, invitrogen, Darmstadt, Germany)) at RT for 1h followed by washing steps and mounting with Rotimount FluorCare DAPI (CarlRoth, Karlsruhe, Germany). Images of the stained sections were done by using a Leica microscope (DM4000M) with twenty-fold magnification. Stained sections were imaged and analysed using FIJI 1.54F software.

Supplementary table 1

Demographics	n = 74
Age – years (mean ± SD)	72.5 ± 13.4
Male Gender – no. (%)	24 (32.4 %)
BMI (kg/m²)	26.55 ± 5.47
**Morbidities**	
Arterial Hypertension - no. (%)	44 (59,5%)
Atrial Fibrillation – no. (%)	36 (48.5%)
Dyslipidemia - no. (%)	14 (18.9%)
Diabetes mellitus - no. (%)	32 (43.2%)
Coronary artery disease - no. (%)	44 (59.5%)
Prior myocardial infarction - no. (%)	10 (13.5%)
Prior PCI – no. (%)	20 (27.0%)
**Medication**	
Aspirin – no. (%)	21 (28.4%)
P2Y12 – no. (%)	9 (12.2%)
OAC – no. (%)	38 (51.4%)
ACE inhibitor – no. (%)	20 (27.0%)
Beta Blockers – no. (%)	50 (67.6%)
Diuretics – no. (%)	41 (55.4%)
PPI – no. (%)	32 (43.2%)
Statins – no. (%)	41 (55.4%)
**Laboratory parameters**	
Platelets	234 ± 82
Hemoglobin (g/dl)	12.59 ± 1.77
Hematocrit (%)	38.7 ± 4.9
Leucocytes	9.08 ± 6.08
GFR (ml/min)	62.96 ± 25.53
CRP (mg/l)	2.16 ± 5.80
LDL (mg/dl)	88.5 ± 32.2
HDL (mg/dl)	51.9 ± 15.4
TG (mg/dl)	105.33 ± 54.4
$HbA1_c$ (%)	6.2 ± 1.1

Table S1: Patient's characteristics. BMI = body mass index, PCI = percutaneous coronary intervention, OAC = oral anticoagulation, PPI = proton pump inhibitor, GFR = glomerular filtration rate, CRP = C-reactive protein, LDL = low-density lipoprotein, HDL = high-density lipoprotein, TG = triglycerides, $HbA1_c$ = glycated hemoglobin

Supplementary figures

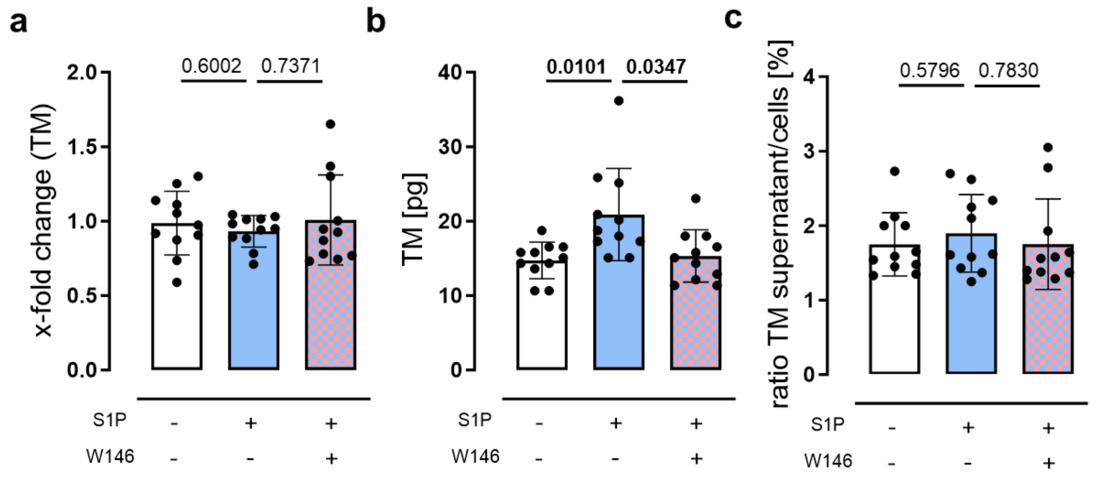

Figure S1: Human umbilical vein endothelial cells (HUVECs) were treated with S1P for 24 hours. **a** S1P treatment showed no effect on TM-mRNA-expression (housekeeping gene: GAPDH). **b** However, soluble TM was increased in HUVEC-supernatant. This was abolished by S1PR1-inhibition with W146. **c** Ratio of soluble supernatant TM and cell-bound TM was unchanged, indication no changes in TM-shedding (n=11, one-way ANOVA followed by Tukey's multiple comparisons test).

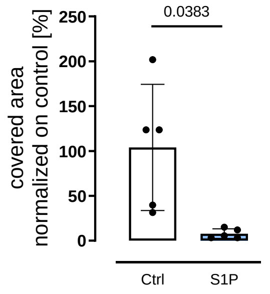

Figure S2: S1P decreased platelet adhesion to HAECs (paired t-test, n=5)

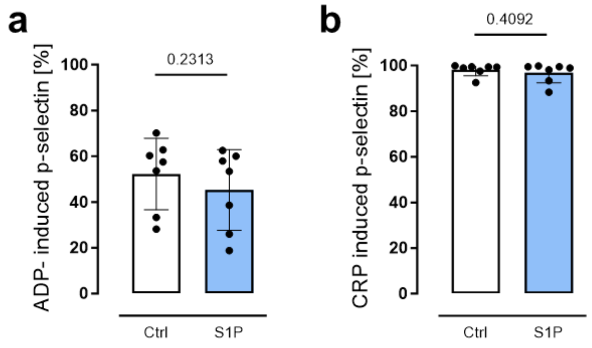

Figure S3: P-selectin-expression on platelets upon activation with **a** adenosine diphosphate (ADP) or **b** collagen-related peptide (CRP) was unchanged after incubation with S1P (n=7, paired t-test).

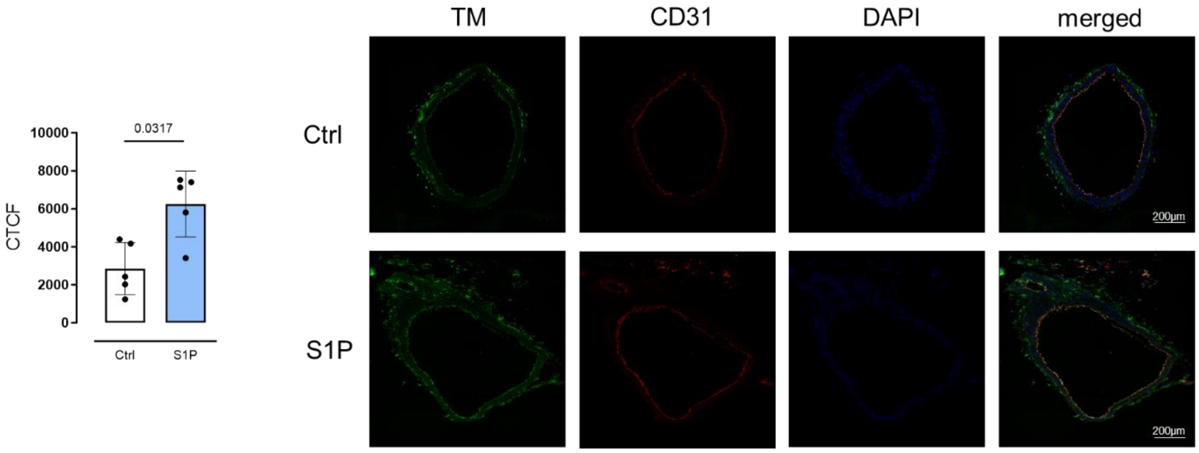

Figure S4: Aortic TM-expression, determined by histological assessment, was increased in C57BL/6J mice that were treated with 38 ng/g S1P 16 hours before (CTCF = Corrected Total Cell Fluorescence, Mann-Whitney test, n=5).

REFERENCES

1. A. Polzin, M. Benkhoff, M. Thienel, M. Barcik, P. Mourikis, K. Shchurovska, C. Helten, V. Ehreiser, Z. Zhe, F. von Wulffen, A. Theiss, S. Peri, S. Cremer, S. Ahlbrecht, S. Zako, L. Wildeis, G. Al-Kassis, D. Metzen, A. Utz, H. Hu, L. Vornholz, G. Pavic, E. Lüsebrink, J. Strecker, S. Tiedt, M. Cramer, M. Gliem, T. Ruck, S. G. Meuth, T. Zeus, C. Mayr, H. B. Schiller, L. Simon, S. Massberg, M. Kelm, T. Petzold, Long-term FXa inhibition attenuates thromboinflammation after acute myocardial infarction and stroke by platelet proteome alteration. *J. Thromb. Haemost.* **23**, 668–683 (2025).

2. M. Valgimigli, H. Bueno, R. A. Byrne, J. P. Collet, F. Costa, A. Jeppsson, P. Juni, A. Kastrati, P. Kolh, L. Mauri, G. Montalescot, F. J. Neumann, M. Petricevic, M. Roffi, P. G. Steg, S. Windecker, J. L. Zamorano, G. N. Levine, 2017 ESC focused update on dual antiplatelet therapy in coronary artery disease developed in collaboration with EACTS: The Task Force for dual antiplatelet therapy in coronary artery disease of the European Society of Cardiology (ESC) and of the European Association for Cardio-Thoracic Surgery (EACTS). *Eur. Heart J.* **39**, 213–260 (2018).

3. R. M'Pembele, S. Ahlbrecht, C. Helten, P. Mourikis, D. Naguib, S. Zako, K. Trojovsky, R. Huhn, T. Petzold, T. Hohlfeld, T. Zeus, M. Kelm, L. Dannenberg, A. Polzin, High on-treatment platelet reactivity: Aspirin versus clopidogrel. *Pharmacology* **108**, 83–89 (2023).

4. M. Benkhoff, A. Polzin, Lipoprotection in cardiovascular diseases. *Pharmacol. Ther.* **264**, 108747 (2024).

5. B. H. Rauch, Sphingosine 1-phosphate as a link between blood coagulation and inflammation. *Cell. Physiol. Biochem.* **34**, 185–196 (2014).

6. Y. Yatomi, F. Ruan, S. Hakomori, Y. Igarashi, Sphingosine-1-phosphate: A platelet-activating sphingolipid released from agonist-stimulated human platelets. *Blood* **86**, 193–202 (1995).

7. D. Nugent, Y. Xu, Sphingosine-1-phosphate: Characterization of its inhibition of platelet aggregation. *Platelets* **11**, 226–232 (2000).

8. N. L. Esmon, R. C. Carroll, C. T. Esmon, Thrombomodulin blocks the ability of thrombin to activate platelets. *J. Biol. Chem.* **258**, 12238–12242 (1983).

9. T. Petzold, M. Thienel, I. Konrad, I. Schubert, R. Regenauer, B. Hoppe, M. Lorenz, A. Eckart, S. Chandraratne, C. Lennerz, C. Kolb, D. Braun, J. Jamasbi, R. Brandl, S. Braun, W. Siess, C. Schulz, S. Massberg, Oral thrombin inhibitor aggravates platelet adhesion and aggregation during arterial thrombosis. *Sci. Transl. Med.* **8**, 367ra168 (2016).

10. T. Sanchez, Sphingosine-1-phosphate signaling in endothelial disorders. *Curr. Atheroscler. Rep.* **18**, 31 (2016).

11. M. Lindner, A. Laporte, L. Elomaa, C. Lee-Thedieck, R. Olmer, M. Weinhart, Flow-induced glycocalyx formation and cell alignment of HUVECs compared to iPSC-derived ECs for tissue engineering applications. *Front. Cell Dev. Biol.* **10**, 953062 (2022).

12. A. Polzin, L. Dannenberg, M. Benkhoff, M. Barcik, C. Helten, P. Mourikis, S. Ahlbrecht, L. Wildeis, J. Ziese, D. Zikeli, D. Metzen, H. Hu, L. Baensch, N. H. Schröder, P. Keul, S. Weske, P. Wollnitzke, D. Duse, S. Saffak, M. Cramer, F. Bönner, T. Müller, M. H. Gräler, T. Zeus, M. Kelm, B. Levkau, Revealing concealed cardioprotection by platelet Mfsd2b-released S1P in human and murine myocardial infarction. *Nat. Commun.* **14**, 2404 (2023).

13. J. G. Garcia, F. Liu, A. D. Verin, A. Birukova, M. A. Dechert, W. T. Gerthoffer, J. R. Bamberg, D. English, Sphingosine 1-phosphate promotes endothelial cell barrier integrity by Edg-dependent cytoskeletal rearrangement. *J. Clin. Invest.* **108**, 689–701 (2001).

14. Y. Zeng, X. H. Liu, J. Tarbell, B. Fu, Sphingosine 1-phosphate induced synthesis of glycocalyx on endothelial cells. *Exp. Cell Res.* **339**, 90–95 (2015).

15. N. Urtz, F. Gaertner, M. L. von Bruehl, S. Chandraratne, F. Rahimi, L. Zhang, M. Orban, V. Barocke, J. Beil, I. Schubert, M. Lorenz, K. R. Legate, A. Huwiler, J. M. Pfeilschifter, C. Beerli, D. Ledieu, E. Persohn, A. Billich, T. Baumruker, M. Mederos y Schnitzler, S. Massberg, Sphingosine 1-phosphate produced by sphingosine kinase 2 intrinsically controls platelet aggregation in vitro and in vivo. *Circ. Res.* **117**, 376–387 (2015).

16. P. Münzer, E. Schmid, B. Walker, A. Fotinos, M. Chatterjee, D. Rath, S. Vogel, S. M. Hoffmann, K. Metzger, P. Seizer, T. Geisler, M. Gawaz, O. Borst, F. Lang, Sphingosine kinase 1 (Sphk1) negatively regulates platelet activation and thrombus formation. *Am. J. Physiol. Cell Physiol.* **307**, C920–C927 (2014).

17. T. E. Wallen, M. Morris, A. Ammann, M. R. Baucom, A. Price, R. Schuster, A. T. Makley, M. D. Goodman, Platelet function is independent of sphingolipid manipulation. *J. Surg. Res.* **300**, 25–32 (2024).

18. S. Mahajan-Thakur, A. Böhm, G. Jedlitschky, K. Schrör, B. H. Rauch, Sphingosine-1-phosphate and its receptors: A mutual link between blood coagulation and inflammation. *Mediators Inflamm.* **2015**, 831059 (2015).

19. A. Polzin, L. Dannenberg, M. Benkhoff, M. Barcik, P. Keul, A. Ayhan, S. Weske, S. Ahlbrecht, K. Trojovsky, C. Helten, S. Haberkorn, U. Flögel, T. Zeus, T. Müller, M. H. Gräler, M. Kelm, B. Levkau, Sphingosine-1-phosphate improves outcome of no-reflow acute myocardial infarction via sphingosine-1-phosphate receptor 1. *ESC Heart Fail.* **10**, 334–341 (2023).

20. A. Polzin, L. Dannenberg, M. Benkhoff, M. Barcik, P. Keul, C. Helten, T. Zeus, M. Kelm, B. Levkau, S1P lyase inhibition starting after ischemia/reperfusion improves postischemic cardiac remodeling. *JACC Basic Transl. Sci.* **7**, 498–499 (2022).

21. F. L. J. Visseren, F. Mach, Y. M. Smulders, D. Carballo, K. C. Koskinas, M. Bäck, A. Benetos, A. Biffi, J. M. Boavida, D. Capodanno, B. Cosyns, C. Crawford, C. H. Davos, I. Desormais, E. Di Angelantonio, O. H. Franco, S. Halvorsen, F. D. R. Hobbs, M. Hollander, E. A. Jankowska, M. Michal, S. Sacco, N. Sattar, L. Tokgozoglu, S. Tonstad, K. P. Tsioufis, I. van Dis, I. C. van Gelder, C. Wanner, B. Williams, 2021 ESC Guidelines on cardiovascular disease prevention in clinical practice. *Eur. Heart J.* **42**, 3227–3337 (2021).

22. J. P. Piccini, M. R. Patel, J. Steffel, K. Ferdinand, I. C. Van Gelder, A. M. Russo, C. S. Ma, S. G. Goodman, J. Oldgren, C. Hammett, R. D. Lopes, M. Akao, R. De Caterina, P. Kirchhof, D. A. Gorog, M. Hemels, M. Rienstra, W. S. Jones, J. Harrington, G. Y. H. Lip, S. J. Ellis, F. W. Rockhold, C. Neumann, J. H. Alexander, T. Viethen, J. Hung, R. Coppolecchia, H. Mundl,

V. Caso, Asundexian versus apixaban in patients with atrial fibrillation. *N. Engl. J. Med.* **392**, 23–32 (2025).

23. M. Benkhoff, P. Mourikis, A. Polzin, Asundexian versus apixaban in patients with atrial fibrillation. *N. Engl. J. Med.* **392**, 1247 (2025).

24. B. Levkau, Cardiovascular effects of sphingosine-1-phosphate (S1P). *Handb. Exp. Pharmacol.* **216**, 147–170 (2013).

25. M. P. McGinley, J. A. Cohen, Sphingosine 1-phosphate receptor modulators in multiple sclerosis and other conditions. *Lancet* **398**, 1184–1194 (2021).

26. Y. Xiong, H. J. Lee, B. Mariko, Y. C. Lu, A. J. Dannenberg, A. S. Haka, F. R. Maxfield, E. Camerer, R. L. Proia, T. Hla, Sphingosine kinases are not required for inflammatory responses in macrophages. *J. Biol. Chem.* **291**, 11465 (2016).

27. M. Schröder, C. Richter, M. H. Juan, K. Maltusch, O. Giegold, G. Quintini, J. M. Pfeilschifter, A. Huwiler, H. H. Radeke, The sphingosine kinase 1 and S1P1 axis specifically counteracts LPS-induced IL-12p70 production in immune cells of the spleen. *Mol. Immunol.* **48**, 1139–1148 (2011).

28. P. Keul, M. M. van Borren, A. Ghanem, F. U. Müller, A. Baartscheer, A. O. Verkerk, F. Stümpel, J. S. Schulte, N. Hamdani, W. A. Linke, P. van Loenen, M. Matus, W. Schmitz, J. Stypmann, K. Tiemann, J. H. Ravesloot, A. E. Alewijnse, S. Hermann, L. J. Spijkers, K. H. Hiller, D. Herr, G. Heusch, M. Schäfers, S. L. Peters, J. Chun, B. Levkau, Sphingosine-1-phosphate receptor 1 regulates cardiac function by modulating Ca^{2+} sensitivity and Na^+/H^+ exchange and mediates protection by ischemic preconditioning. *J. Am. Heart Assoc.* **5**, e003393 (2016).

29. G. Theilmeier, C. Schmidt, J. Herrmann, P. Keul, M. Schäfers, I. Herrgott, J. Mersmann, J. Larmann, S. Hermann, J. Stypmann, O. Schober, R. Hildebrand, R. Schulz, G. Heusch, M. Haude, K. von Wnuck Lipinski, C. Herzog, M. Schmitz, R. Erbel, J. Chun, B. Levkau, High-density lipoproteins and their constituent, sphingosine-1-phosphate, directly protect the

heart against ischemia/reperfusion injury in vivo via the S1P3 lysophospholipid receptor. *Circulation* **114**, 1403–1409 (2006).

30. N. Percie du Sert, V. Hurst, A. Ahluwalia, S. Alam, M. T. Avey, M. Baker, W. J. Browne, A. Clark, I. C. Cuthill, U. Dirnagl, M. Emerson, P. Garner, S. T. Holgate, D. W. Howells, N. A. Karp, S. E. Lazic, K. Lidster, C. J. MacCallum, M. Macleod, E. J. Pearl, O. H. Petersen, F. Rawle, P. Reynolds, K. Rooney, E. S. Sena, S. D. Silberberg, T. Steckler, H. Würbel, The ARRIVE guidelines 2.0: Updated guidelines for reporting animal research. *Br. J. Pharmacol.* **177**, 3617–3624 (2020).

31. P. Mourikis, M. Benkhoff, L. Wildeis, M. Barcik, C. Helten, C. Coman, F. A. Solari, D. Krahn, L. Dannenberg, S. Ahlbrecht, D. Zikeli, A. Utz, K. Trojovsky, H. Richter, G. Al Kassis, R. M'Pembele, S. Zako, T. Huckenbeck, S. Bauer, D. Schmitz, S. Pfeiler, N. Gerdes, C. Dücker, J. Pircher, Z. Zhe, M. Thienel, Q. Ul Ain, P. Keul, N. Kirkby, D. Sohn, W. Budach, T. Hohlfeld, K. Schrör, B. Levkau, T. Zeus, S. H. L. Verhelst, R. Ahrends, A. Sickmann, J. Mitchell, S. Mora, J. E. Manson, D. L. Bhatt, U. Landmesser, S. Massberg, M. Kelm, T. Petzold, A. Polzin, Icosapent ethyl reduces arterial thrombosis by inhibition of cyclooxygenase-1-induced platelet reactivity. *Sci. Transl. Med.* **17**, eado0610 (2025).

32. B. Peng, S. Geue, C. Coman, P. Munzer, D. Kopczynski, C. Has, N. Hoffmann, M. C. Manke, F. Lang, A. Sickmann, M. Gawaz, O. Borst, R. Ahrends, Identification of key lipids critical for platelet activation by comprehensive analysis of the platelet lipidome. *Blood* **132**, e1–e12 (2018).

33. K. J. Adams, B. Pratt, N. Bose, L. G. Dubois, L. St John-Williams, K. M. Perrott, K. Ky, P. Kapahi, V. Sharma, M. J. MacCoss, M. A. Moseley, C. A. Colton, B. X. MacLean, B. Schilling, J. W. Thompson, Skyline for Small Molecules: A Unifying Software Package for Quantitative Metabolomics. *J. Proteome Res.* **19**, 1447–1458 (2020).
